# Sirolimus for epileptic seizures associated with focal cortical dysplasia type II

**DOI:** 10.1002/acn3.51505

**Published:** 2022-01-18

**Authors:** Mitsuhiro Kato, Akiko Kada, Hideaki Shiraishi, Jun Tohyama, Eiji Nakagawa, Yukitoshi Takahashi, Tomoyuki Akiyama, Akiyoshi Kakita, Noriko Miyake, Atsushi Fujita, Akiko M. Saito, Yushi Inoue

**Affiliations:** ^1^ Department of Pediatrics Showa University School of Medicine Tokyo Japan; ^2^ Clinical Research Center National Hospital Organization Nagoya Medical Center Nagoya Japan; ^3^ Department of Pediatrics Hokkaido University Hospital Sapporo Japan; ^4^ Department of Child Neurology National Hospital Organization Nishiniigata Chuo Hospital Niigata Japan; ^5^ Department of Child Neurology, National Center Hospital National Center of Neurology and Psychiatry Tokyo Japan; ^6^ National Hospital Organization Shizuoka Institute of Epilepsy and Neurological Disorders Shizuoka Japan; ^7^ Department of Child Neurology Okayama University Graduate School of Medicine, Dentistry and Pharmaceutical Sciences Okayama Japan; ^8^ Department of Pathology, Brain Research Institute Niigata University Niigata Japan; ^9^ Department of Human Genetics Yokohama City University Graduate School of Medicine Yokohama Japan; ^10^ Department of Human Genetics Research Institute National Center for Global Health and Medicine Tokyo Japan

## Abstract

**Objective:**

To determine whether sirolimus, a mechanistic target of rapamycin (mTOR) inhibitor, reduces epileptic seizures associated with focal cortical dysplasia (FCD) type II.

**Methods:**

Sixteen patients (aged 6–57 years) with FCD type II received sirolimus at an initial dose of 1 or 2 mg/day based on body weight (FCDS‐01). In 15 patients, the dose was adjusted to achieve target trough ranges of 5–15 ng/mL, followed by a 12‐week maintenance therapy period. The primary endpoint was a lower focal seizure frequency during the maintenance therapy period. Further, we also conducted a prospective cohort study (RES‐FCD) in which 60 patients with FCD type II were included as an external control group.

**Results:**

The focal seizure frequency reduced by 25% in all patients during the maintenance therapy period and by a median value of 17%, 28%, and 23% during the 1–4‐, 5–8‐, and 9–12‐week periods. The response rate was 33%. The focal seizure frequency in the external control group reduced by 0.5%. However, the background characteristics of external and sirolimus‐treated groups differed. Adverse events were consistent with those of mTOR inhibitors reported previously. The blood KL‐6 level was elevated over time.

**Interpretation:**

The reduction of focal seizures did not meet the predetermined level of statistical significance. The safety profile of the drug was tolerable. The potential for a reduction of focal seizures over time merit further investigations.

## Introduction

Fewer than 20% of patients with focal cortical dysplasia (FCD) have transient responsiveness to pharmacotherapy,^1^ and most require surgical resection of the brain lesion. However, epilepsy surgery for FCD is one of the most challenging procedures because lesions are commonly located in the functional cortical area and have an uncertain demarcation in the epileptogenic zone on magnetic resonance imaging (MRI) and electroencephalogram (EEG).^2^ Approximately one‐third of patients still present with seizures after surgical resection with comprehensive neuroimaging and electrophysiological evaluations.^3^

Somatic mutations in the mechanistic target of rapamycin (*MTOR*) and other genes correlated with the mTOR signaling pathway, such as *AKT3* and *PIK3CA*, have been identified in the pathological brain tissues of patients with FCD type IIa or IIb.^4^, ^5^, ^6^, ^7^, ^8^, ^9^ *MTOR* is the most frequent causative gene for FCD IIa or IIb and the second common causative gene for hemimegalencephaly (Table S1). These mutations cause the elevated activation of mTOR signaling, which is a major cause of FCD type II.^4^, ^5^, ^10^ The intrinsic epileptogenicity of FCD itself has been confirmed via intraoperative electrocorticography and stereo‐EEG.^11^, ^12^ Sirolimus or rapamycin is an mTOR inhibitor, which has suppressed epileptic seizures in an FCD model of mice with hyperactivated mutant mTOR.^4^ This drug is beneficial for the treatment of seizures in animal models of genetic mTOR hyperactivation and in patients with tuberous sclerosis complex (TSC) caused by *TSC2* or *TSC1* mutation leading to mTOR hyperactivation.^13^, ^14^ Additionally, reduction in seizure frequency has been reported in a patient with hemimegalencephaly due to a somatic mosaic *MTOR* mutation (Table S2).^15^ We conducted a single‐center clinical trial (as a proof‐of‐concept study) to validate the effect of sirolimus on seizure control in a small number of patients with FCD type II in 2018. Results showed that sirolimus was beneficial for treating seizures in patients with FCD (manuscript submitted). Herein, we report the results of the first single‐arm, open‐label, multicenter clinical trial that assessed the efficacy and safety of sirolimus for the treatment of epileptic seizures in patients with FCD type II (FCDS‐01).

## Methods

## Study design and participants

We conducted this investigator‐initiated, single‐arm, open‐label, multicenter trial at five institutions in Japan between December 2018 and August 2020. The trial rationale and design have been described previously.^16^

**Figure 1:**
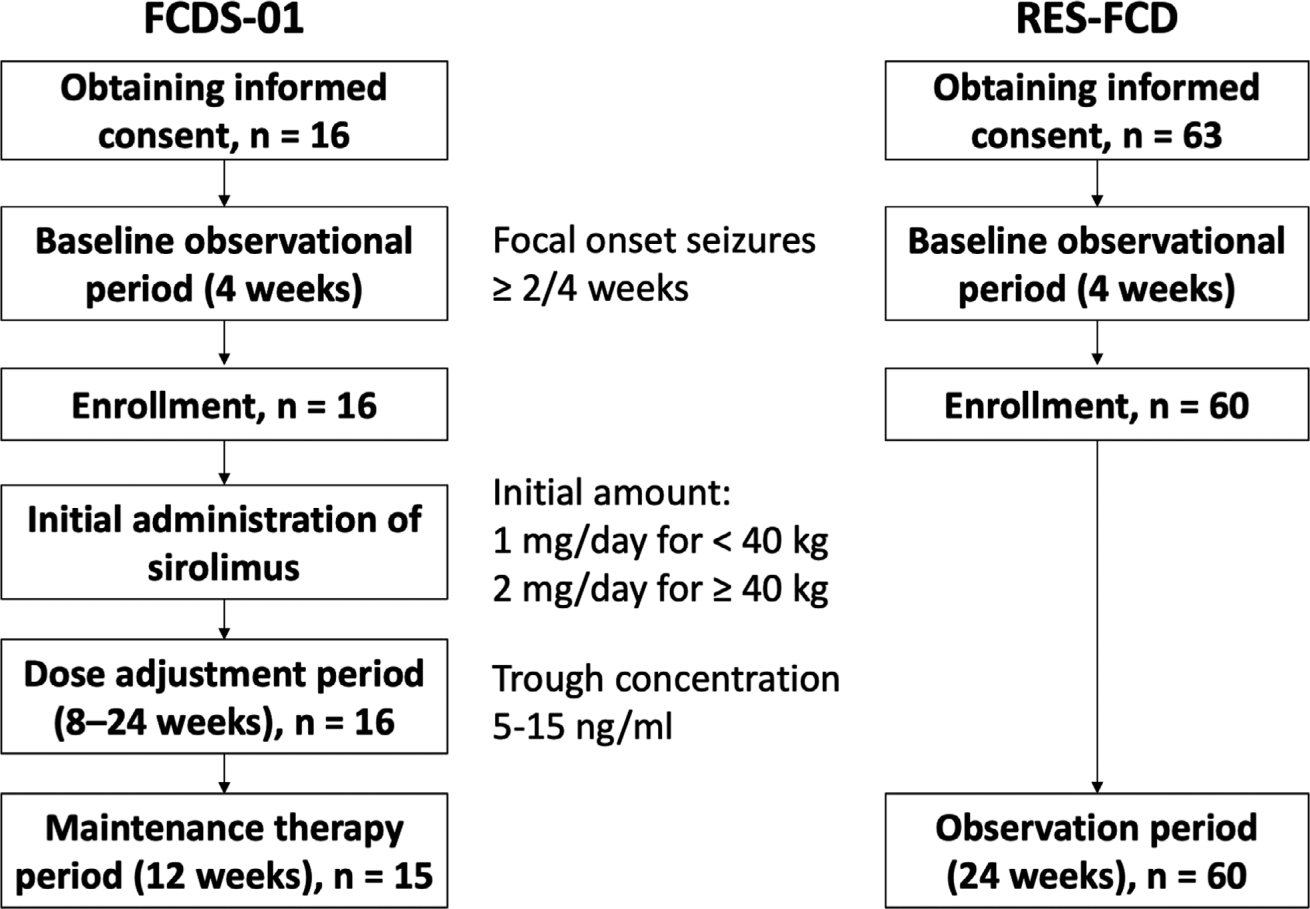
Schematic flow of the trial design of FCDS‐01 and RES‐FCD.

Patients aged 6–65 years who were diagnosed with FCD type II via brain MRI in accordance with the radiological criteria^17^ or histopathological findings were eligible.^18^ All patients had focal onset seizures, including focal to bilateral tonic–clonic seizure, at a frequency of more than twice in a 28‐day baseline phase (Fig. 1). All patients had a treatment history of more than two antiepileptic drugs for at least 52 weeks after epilepsy diagnosis and were receiving one to four concomitant antiepileptic drugs. The dose and regimen of concurrent antiepileptic drugs were sustained from the 8th week before study enrollment. We excluded patients with a history of undergoing neurosurgical procedures within 28 weeks prior to enrollment with consideration of other treatment protocols including antiepileptic drugs.

### Standard protocol approvals, registrations, and patient consents

The research protocol was approved by the central ethics review committee for clinical research of the National Hospital Organization and the institutional review board of each institution (FCDS‐01). An independent data and safety monitoring board reviewed the trial progress. Written informed consent was obtained from all patients or their legal guardians prior to study enrollment. Able patients under 16 years provided consent. This study was registered at the University Hospital Medical Information Network Clinical Trial Registry (UMIN000033504).

### Study medication and procedures

Patients with a body weight of <40 and ≥40 kg received oral sirolimus as a tablet at an initial dose of 1 and 2 mg/day, respectively, once a day. The dose was adjusted to achieve the target blood trough levels (range: 5–15 ng/mL) during the 8–24‐week titration period (dose‐adjustment phase). If the concentrations were <5 ng/mL, the daily sirolimus dosage was increased by 1 mg every 2 weeks until the 8th week, and then every 4 weeks until the 24th week. From the time when the trough concentration reached the target range, or after 24 weeks if the trough concentration did not reach the target range, 12‐week maintenance therapy was started. The participants visited the hospitals at 4, 8, and 12 weeks during the maintenance therapy period. No further adjustments were made during this period unless medication‐related adverse events required treatment, discontinuation, or dosage adjustment to half of the current dose.

### Trial outcomes

The primary endpoint was a reduced frequency of focal seizures (including focal to bilateral tonic–clonic seizures), which is a main seizure type in patients with FCD, per 28 days during the maintenance therapy period. Patients or their guardians recorded data in a seizure diary throughout the study. The secondary endpoints were changes in the frequency of generalized seizures, epileptic spasms, and status epilepticus; response rate, which is the percentage of patients showing a ≥50% reduction in the frequency of focal seizure during the maintenance therapy period; proportion of patients who are free from focal seizure during the maintenance therapy period; decreased incidence and response rates of focal seizures at 4, 8, and 12 weeks in the maintenance therapy period; and adverse events. During the study, adverse events were recorded and categorized based on the Common Terminology Criteria for Adverse Events version 4.03. All investigators completed the Columbia‐Suicide Severity Rating Scale training^19^ prior to the study.

We performed an exploratory investigation of the pharmacokinetics of sirolimus, correlation between combination drugs and pharmacokinetics of sirolimus, degree of decrease in seizure frequency using FCD registration data as the external control group, and changes in blood KL‐6 level, which is a chemical marker for pneumonitis (reference range: <500 U/mL),^20^ and white blood cell and lymphocyte counts at each visit during dose‐adjustment period and at 12 weeks in the maintenance therapy period. Blood was also collected at 4 and 8 weeks in the maintenance therapy period if necessary. Next, a prospective cohort study on epileptic seizures associated with FCD type II (RES‐FCD, UMIN000033606) was conducted to compare the frequency of seizures between the control and clinical sirolimus‐treated groups from August 2018 to September 2020. The same patient selection criteria of the clinical trial (FCDS‐01) were adopted to external cohort (RES‐FCD). The primary endpoint of RES‐FCD was a reduced frequency of focal seizures per 28 days during a 24‐week observation period. The secondary endpoints of RES‐FCD were similar to those of the clinical trial. The blood testosterone concentrations of male participants aged older than 9 years were investigated in the middle of the study due to the risk of sirolimus‐associated male infertility.^21^

### Statistical analyses

The sample size was 15, which was based on the feasibility of the study. The study was registered within the given period even if the sample size exceeded 15. To ensure efficacy, two analysis sets were used: full analysis set (FAS) and per protocol set (PPS). FAS was the main analysis set, and it comprised patients who were enrolled and treated with the drug. However, patients who had serious protocol violations, such as the absence of FCDS type II diagnosis or informed consent, and who were not eligible and without any data about seizure after drug administration were excluded from the FAS. Meanwhile, the PPS comprised patients from the FAS who had no serious protocol violation, met the provision of the clinical trial practice plan, and could be evaluated for efficacy according to the protocol. As for the reduction rate of focal seizures, the Wilcoxon one‐sample signed‐rank test was used to evaluate the null hypothesis against a median of 0. Moreover, the log‐transformed incidence of focal seizures, ratio of the maintenance therapy period to baseline, and 90% confidence interval (CI) were calculated. The response rate and proportion of patients who experienced focal seizure resolution and their 90% CI were examined. In addition, the frequency of focal seizures at baseline and at 4, 8, and 12 weeks was evaluated via a regression analysis with time as a fixed effect and the patient as a random effect, which was based on a negative binomial distribution with the response variable as the frequency of seizures (times/28 days) and the log‐transformed observation period as the offset variable. To examine differences in characteristics between the external control and sirolimus‐treated groups, the Fisher's exact test and Wilcoxon rank‐sum test were used. The propensity score of the groups (sirolimus‐treated/control) based on each characteristic was assessed. For the subgroup analysis, we calculated the rate of reduction of focal seizure frequency in patients who did and did not undergo surgery. In addition, changes from baseline in KL‐6 were assessed using the one‐sample Wilcoxon test. All statistical analyses were conducted using the SAS version 9.4 (SAS Institute, Cary, NC, USA).

## Results

### Patients

Sixteen patients (aged 7–57 years) were screened, and none dropped out during the baseline phase. One patient (Patient 13) dropped out during the dose‐adjustment phase due to the use of prohibited drugs (midazolam and thiopental sodium) for status epilepticus, which had occurred repeatedly before administration of sirolimus. This patient had no data on seizure frequency during the maintenance therapy period; thus, the data were eliminated from the efficacy analysis in FAS. Further, two more patients were excluded from the FAS due to non‐compliance and use of prohibited drugs (PPS, *n* = 13). However, they were included in the safety analysis. Table 1 shows the demographic and baseline characteristics of individual patients (*n* = 16). The patient's mean age was 16.7 ± 12.1 years (mean ± standard deviation [SD]), and the median age was 13 years. In addition, 13 patients underwent neurosurgery; six and seven of these patients were classified as FCD type IIa and FCD type IIb, respectively. Three patients who did not undergo neurosurgery presented with FCD type II, which were classified according to brain MRI findings. Patient 11 who had a pathogenic variant of *MTOR* (NM_004958:c.4448G>A:p.Cys1483Tyr) with a variant allele frequency of 4% in the surgically resected brain tissue atypically experienced her first seizure at 53 years of age. Focal seizure types were classified as focal awareness seizure (*n* = 7), focal impaired awareness seizure (*n* = 12), and focal to bilateral tonic–clonic seizure (*n* = 4). The daily dose during the maintenance therapy period was 2 or 4 mg (Table 2). In three patients, the sirolimus dose was modified during the maintenance period due to appetite loss and somnolence (Patient 4), pneumonitis (Patient 9), and proteinuria (Patient 12). The blood concentrations of sirolimus (mean ± SD) at the 4‐, 8‐, and 12‐week maintenance therapy periods were 5.0 ± 2.1, 4.6 ± 2.6, and 5.0 ± 1.9 ng/mL, respectively. Meanwhile, the concentrations at the dose‐adjustment phase and maintenance therapy period did not differ between a combination with and without each concomitant antiepileptic drug.

**Table 1:** Baseline characteristics of the 16 patients receiving sirolimus.

Patient No.	Age at study entry (years)	Sex	FCD type	Age at seizure onset (years)	Focal seizure type	Seizure type other than focal seizure	Neurological findings	Cognitive dysfunction
1	11	M	IIa	0	FIAS			ID
2	9	M	IIb	4	FAwS			ADHD
3	21	F	IIa	0		Tonic, myoclonic	Hemiplegia, involuntary movement, sensory disturbance	ID
4	6	F	IIb	0	FIAS		Hemiplegia	ID
5	21	F	II	14	FIAS			
6	13	M	IIa	0	FAwS, FIAS			ID, personality disorder
7	24	F	IIb	0	FIAS	Tonic, ES	Hemiplegia	ID
8	7	M	IIa	1	FAwS, FBTC	Tonic		ID, ASD
9	15	M	IIa	7	FAwS, FIAS			Tics
10	12	F	IIa		FAwS, FIAS, FBTC		Hemiplegia	ID
11	57	F	IIb	53	FIAS	Tonic–clonic	Sensory disturbance	
12	12	M	II	0	FIAS			ID, ASD
13	13	F	IIb	0	FIAS	ES	Paraplegia, able to sit alone	ID
14	7	F	IIb	1	FAwS, FIAS, FBTC	ES	Hemiplegia	
15	20	M	II	9	FAwS, FBTC			ID
16	19	M	IIb	1	FIAS	Tonic		ID

ADHD, attention‐deficit/hyperactivity disorder; ASD, autistic spectrum disorder; ES, epileptic spasms; F, female; FAwS, focal awareness seizure; FBTC, focal to bilateral tonic–clonic seizure; FCD, focal cortical dysplasia; FIAS, focal impaired awareness seizure; ID, intellectual disability; M, male.

**Table 2:** Daily sirolimus dose during the maintenance therapy period, blood concentrations of sirolimus, and frequency and reduction rate of focal seizures in each patient.

Patient no.	Daily dose during maintenance therapy (mg)	Blood concentration of sirolimus at 4, 8, and 12 weeks of maintenance therapy (ng/mL)	Focal seizure frequency at baseline (per 28 days)	Focal seizure frequency during maintenance therapy (per 28 days)				Reduction rate of focal seizures (%)			
				0–4 weeks	5–8 weeks	9–12 weeks	0–12 weeks	0–4 weeks	5–8 weeks	9–12 weeks	0–12 weeks
1	4	5.0/7.3/6.1	78.2	80	66	60.9	69.5	−2.29	15.61	22.17	11.17
2	4	−/−/2.5	10.6	20	18	16.8	18.2	−88.31	−69.48	−58.18	−70.93
3	4	−/−/4.5	493.3	184.8	242.3	254.9	226.3	62.54	50.88	48.33	54.12
4	4 (2 at 12 weeks)	−/6.3/5.0	24	27	6	0.8	10.6	−12.5	75	96.57	55.93
5	2	2.4/1.9/2.3	12	7.2	7	8	7.4	40	41.67	33.33	38.46
6	4	2.9/2.9/3.0^1^	104	110.4	99.2	101.3	103.6	−6.11	4.62	2.6	0.41
7	4	6.8/−/6.7	1122	771.2	804.8	1079.7	867.4	31.27	28.27	3.77	22.69
8	4	−/−/7.7	4	6.7	3.2	0	3	−66.67	20	100	25
9	4 (2 at 9 weeks)	−/−/4.4	22	54	18.9	57	41.5	−145.45	13.9	−159.26	−88.76
10	2	−/−/3.1	78	30	21.8	34	28.7	61.54	72.08	56.41	63.24
11	2	7.7/−/7.0	4.9	2	2	0	1.6	59.52	59.52	100	67.62
12	2	−/−/3.7	33.8	28	32.8	34.4	31.6	17.14	2.86	−1.69	6.52
13	–		50.2	–	–	–	–	–	–	–	–
14	4	−/−/5.0	5	4.1	3	3.9	3.7	17.04	40	22.76	26.67
15	2	5.2/−/8.0	4	5	4	5.3	4.7	−25	0	−33.33	−18.18
16	4	−/−/6.4	26.2	0	0	0	0	100	100	100	100

^1^ One day after the end of week 12.

### Efficacy

**Figure 2:**
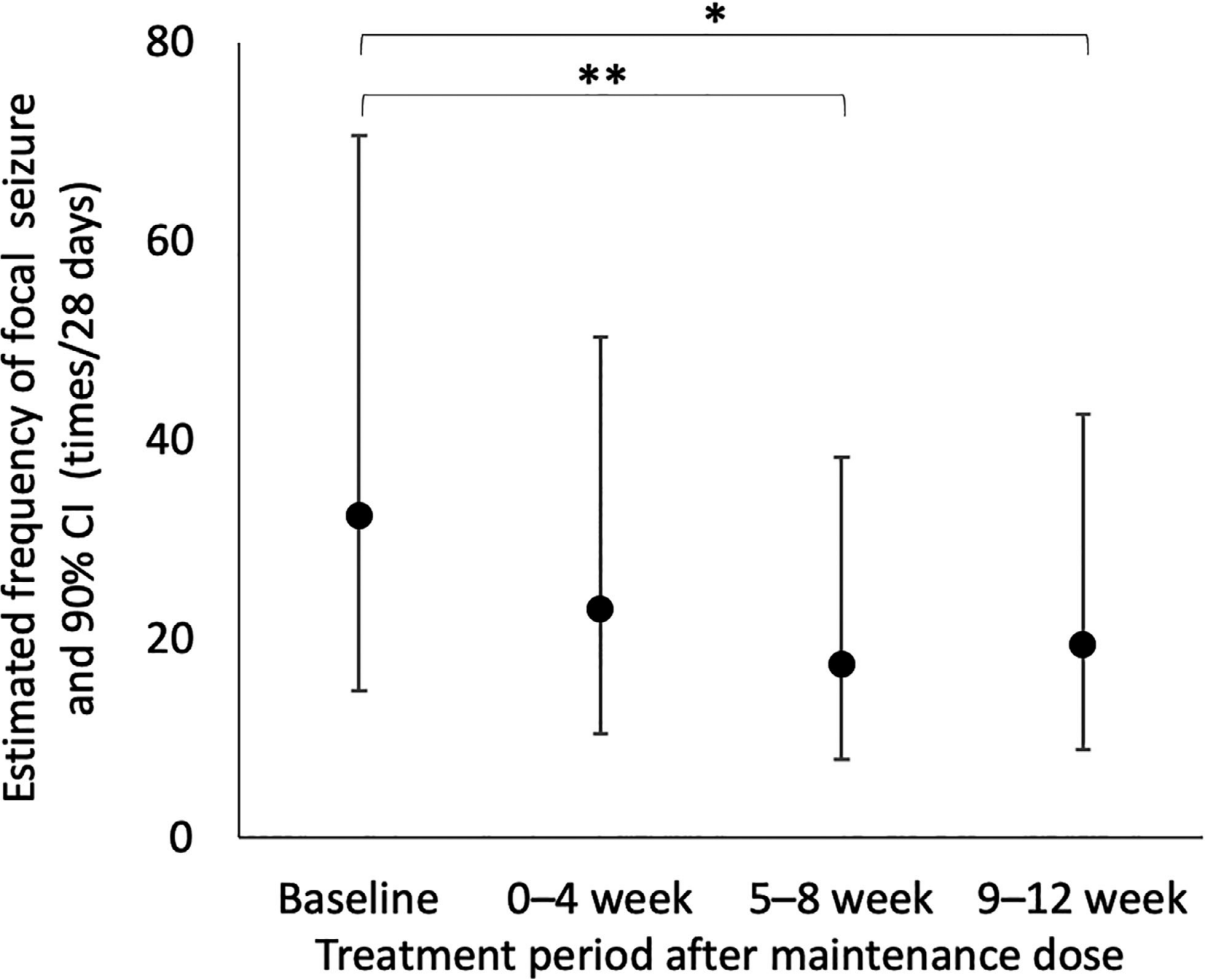
Cumulative effect of sirolimus on focal seizure frequency over time. *p = 0.013, **p = 0.003.

The median frequency of focal seizures per 28 days at baseline and the maintenance therapy period in the FAS were 24.0 (range: 4.0–1122.0) and 18.2 (range: 0–867.4), respectively. The median reduction rate of focal seizure frequency per 28 days during the maintenance therapy period was 25% (range: −89% to 100%), which was not a substantial change (*p* = 0.11). The estimated frequency of focal seizures per 28 days were 32.3 (90% CI: 14.8–70.7) at baseline, 23.0 (90% CI: 10.5–50.3) at 1–4 week, 17.4 (90% CI: 7.9–38.2) at 5–8 week, and 19.4 (90% CI: 8.8–42.5) at 9–12 week (Fig. 2). The frequency of focal seizure at the 5–8‐ and 9–12‐week maintenance therapy periods decreased based on the regression analysis (*p* = 0.003 and 0.013, respectively). Similar results were observed in the PPS. The median frequency of focal seizures per 28 days at baseline and during the maintenance therapy period were 22.0 (range, 4.0–1122.0) and 18.2 (range, 0–867.4), respectively, in the PPS. The median reduction rate of focal seizure frequency per 28 days during the maintenance therapy period was 25% (range, −89% to 100%, *p* = 0.19). The response rate was 33% (5/15, 90% CI: 14–58%). One patient (Patient 16) was free from seizure during the maintenance therapy period (7%, 90% CI: 0.3–28%). The frequency of generalized seizures per 28 days (mean ± SD) at baseline and during the maintenance therapy period were 5.9 ± 15.69 (range: 0–47) and 2.5 ± 9.3 (range: 0–36), respectively. None of the patients showed epileptic spasms or status epilepticus at baseline or during the maintenance therapy period.

### Comparison between the external control and sirolimus‐treated groups

**Figure 3:**
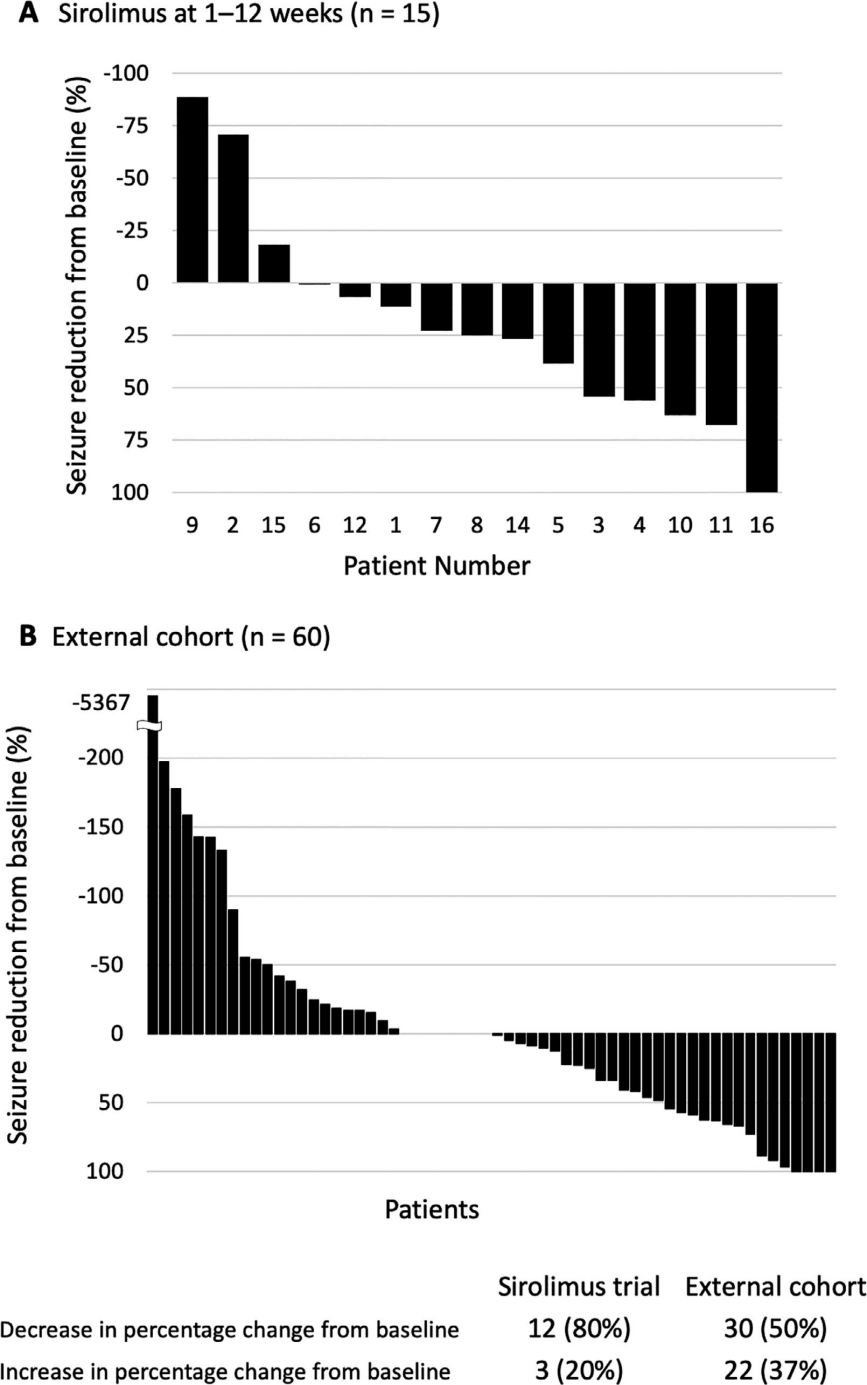
Percentage change in the frequency of focal seizures from baseline. Each bar represents one patient in the sirolimus‐treated group (A) and the external control group (B).

Figure 3 shows changes in seizure frequency in each participant of the external control and sirolimus‐treated groups. The distribution of background characteristics of the external control group (RES‐FCD) versus the sirolimus‐treated group differed (Table 3). In the two groups, sampling bias (*p* < 0.15) affected age and frequency of focal seizures, epileptic spasms, sensory disturbance, and surgical intervention. The propensity score based on age, age at disease onset, baseline frequency of focal seizures, and surgery differed between the external control and sirolimus‐treated groups (median [interquartile range] of 0.02 [0.01–0.16] and 0.67 [0.32–0.77]). Because comparability could not be guaranteed, we did not estimate the difference in the reduction rate of focal seizure frequency between the two groups. However, the median reduction rates for the external control and sirolimus‐treated groups were 0.5% and 25%, respectively. In the subgroup analysis of patients who did and did not undergo surgery, the median rate of reduction of focal seizure frequency for the external control and sirolimus‐treated groups were 0% (*n* = 19) and 25% (*n* = 13) in patients who underwent surgery and 5% (*n* = 41) and 10% (*n* = 2) in patients who did not undergo surgery, respectively.

**Table 3:** Comparison of demographic characteristics between the external control and sirolimus‐treated groups.

Number of cases	Controls	FCDS‐01	*p* value
	*n* = 60	*n* = 15	
Sex			
Male	25 (42%)	8 (53%)	0.56
Female	35 (58%)	7 (47%)	
Age (years)			
Average ± standard deviation	23.9 ± 13.51	16.9 ± 12.49	0.04
Median	22.5	13	
Minimum–maximum	6–59	6–57	
The age of onset			
Average ± standard deviation	5.7 ± 5.88	6.4 ± 14.08^1^	0.28
Median (minimum–maximum)	3 (0–20)	1 (0–53)	
Seizure type			
Focal awareness seizure	24 (40%)	7 (47%)	0.77
Focal impaired awareness seizure	40 (67%)	12 (80%)	0.37
Focal to bilateral tonic–clonic seizure	28 (47%)	6 (40%)	0.78
Myoclonic seizure	0 (0%)	1 (7%)	0.20
Tonic seizure	8 (13%)	4 (27%)	0.24
Tonic–clonic seizure	7 (12%)	1 (7%)	1.00
Epileptic spasms	1 (2%)	2 (13%)	0.10
Seizure frequency			
Baseline frequency of focal seizures per 28 days	22.2 ± 41.81	134.8 ± 299.51	0.03
Neurological findings			
Hemiplegia	11 (18%)	5 (33%)	0.36
Quadriplegia	2 (3%)	0 (0%)	1.00
Sensory disturbance	0 (0%)	2 (13%)	0.04
Ataxia	2 (3%)	0 (0%)	1.00
Involuntary movement	1 (2%)	1 (7%)	0.36
Cognitive dysfunction			
Intellectual disability	30 (50%)	10 (67%)	0.39
ASD	8 (13%)	2 (13%)	1.00
ADHD	0 (0%)	1 (7%)	0.40
Others	1 (2%)	2 (13%)	0.20
Treatments other than sirolimus			
Pharmacotherapy	60 (100%)	15 (100%)	–
ACTH	3 (5%)	2 (13%)	0.26
Surgery	19 (32%)	13 (87%)	<0.001
Lesionectomy/lobectomy	17 (28%)	9 (60%)	–
Multilobectomy	1 (2%)	4 (27%)	–
Callosotomy	0 (0%)	2 (13%)	–
Vagus nerve stimulation	1 (2%)	2 (13%)	–

The *p* value was calculated using the Wilcoxon signed‐rank test for sex and the Fisher's exact test for others. ACTH, adrenocorticotropic hormone; ADHD, attention‐deficit/hyperactivity disorder; ASD, autistic spectrum disorder; *n*, number.

^1^ The age of seizure onset was unknown in Patient 10, who was then excluded.

### Safety

**Figure 4:**
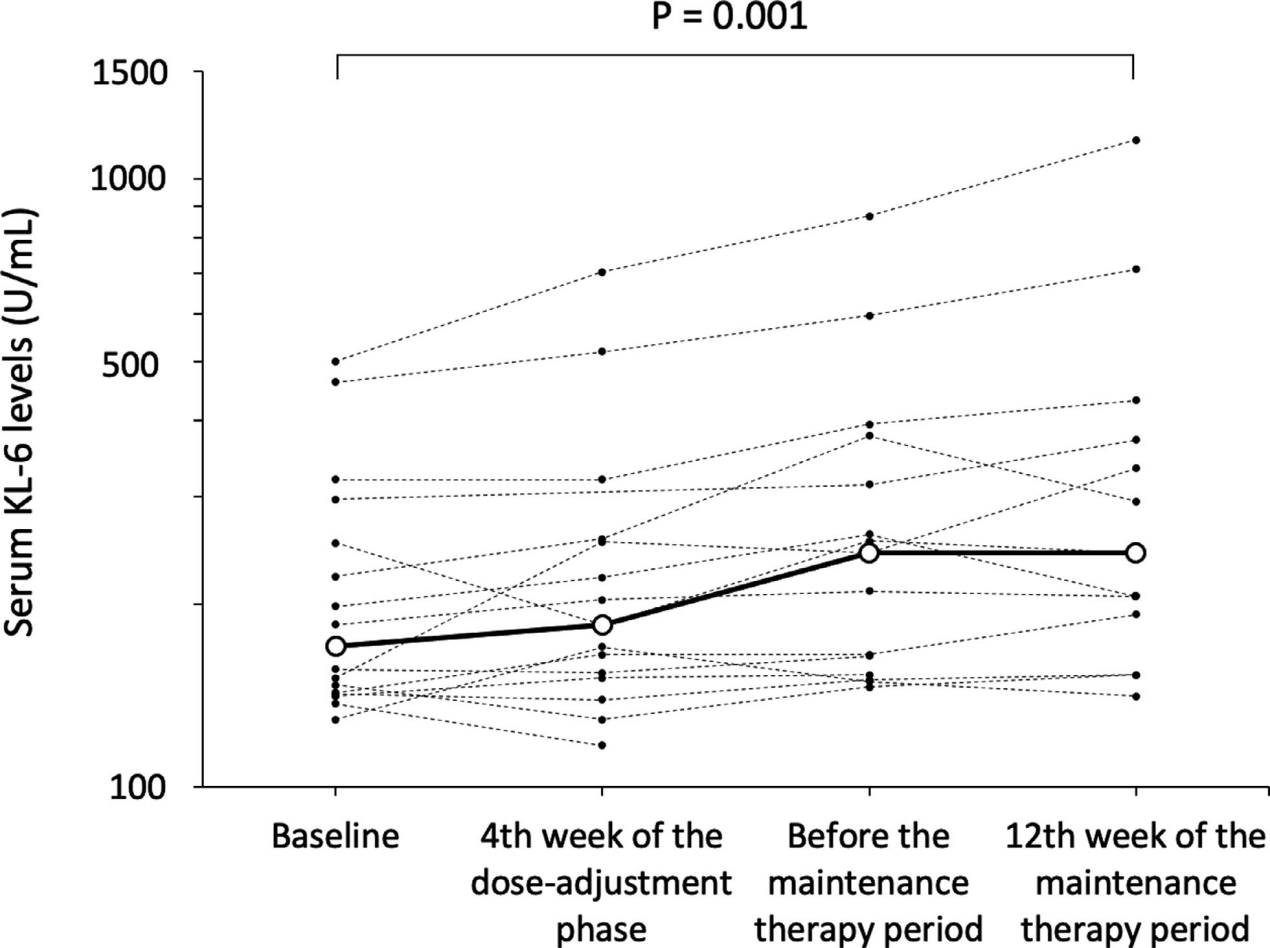
Blood concentration of KL‐6 in patients treated with sirolimus. The small dots indicate each measurement of blood KL‐6 concentration, and the broken lines connect each patient. The circles connected with a bold line indicate the median value of blood KL‐6 concentration.

All patients presented with adverse events (Table 4, Table S3). Three patients experienced severe adverse events, such as epileptic seizure (twice in Patient 10), head skin tear due to head trauma associated with seizure attack (Patient 11), and gastroenteritis due to Norovirus infection at the 9th day of dose‐adjustment phase and status epilepticus at the 44th day of dose‐adjustment phase (Patient 13). All severe adverse events were supposed to be irrelevant for sirolimus, and the patients completely recovered. The median blood KL‐6 level (U/L) was 170.5 (*n* = 16, range: 129–501) at baseline, and it increased to 185 (*n* = 15, range: 117–702, *p* = 0.094), 243 (*n* = 15, range: 146–867, *p* < 0.001), and 243 (*n* = 13, range: 141–1158, *p* = 0.001) at week 4 of the dose‐adjustment phase, just before the maintenance therapy period and at week 12 of the maintenance therapy period, respectively (Fig. 4). Patient 3 and 7 had KL‐6 above 500 U/mL. However, they did not develop pneumonitis. Patient 9 had grade 2 pneumonitis during the maintenance therapy period. Thus, treatment with sirolimus was discontinued for 4 days, during which the KL‐6 levels increased from 320 at baseline to 432 at week 12 of the maintenance therapy period.

**Table 4:** Adverse events reported in two or more patients receiving sirolimus.

Category	Total	Grade 1	Grade 2	Grade 3
Total	16	14	9	3
Gastrointestinal disorders	11	8	4	0
Stomatitis	11	7	4	0
Infections	9	7	4	1
Pharyngitis	6	5	1	0
Gastroenteritis	2	1	0	1
Skin and subcutaneous tissue disorders	7	6	2	0
Dermatitis	2	2	0	0
Nervous system disorders	6	3	2	2
Headache	2	1	1	0
Respiratory, thoracic, and mediastinal disorders	3	3	2	0
Nasal drip	2	1	1	0

## Discussion

In this trial, the reduction of focal seizures did not meet the predetermined level of statistical significance. However, focal seizure frequency reduced over time in sirolimus‐treated patients. The efficacy of this drug in patients with FCD type II (response rate, 33.3%) was comparable to that of everolimus, another mTOR inhibitor, for treatment‐resistant seizures in patients with TSC.^22^

The average trough level of sirolimus was relatively lower than the anticipated value during the maintenance therapy period. There was no difference in terms of trough levels between five responders (5.2 ± 1.6 ng/mL) and 10 nonresponders (4.9 ± 2.1 ng/mL). Everolimus had a dose or blood level‐dependent effect against seizure in patients with TSC, with a response rate of 28.2% for a low‐trough level (3–7 ng/mL) and 40.0% for a high‐trough level (8–15 ng/mL).^23^ The administration of sirolimus at a higher dose might be beneficial for reducing seizure frequency in patients with FCD type II.

We included an FCD cohort (RES‐FCD) and collected clinical information from 60 patients who were included in the control group. The two groups differed in terms of background characteristics, thereby affecting the prognosis of seizures and comorbidities.^23^, ^24^ More frequent focal seizures in the sirolimus‐treated group suggest a higher disease severity than that in the control group.

All patients developed adverse events during the study period. The safety profile of this study was consistent with that of previous studies of patients treated with sirolimus or everolimus.^25^, ^26^ Stomatitis was the most common adverse event as known well. The frequency of stomatitis is gradually decreasing over time,^27^ and none of our patients required discontinuation of treatment in this short‐term trial. The second most frequent adverse event was infection, and it is the most common cause of treatment discontinuation in severe cases.^28^ Notably, pneumonitis is a serious side effect of mTOR inhibitors.^29^ The blood KL‐6 level was elevated over time. Hence, patients treated with sirolimus must be continually monitored.

Only one patient underwent mutation analysis. Somatic activating mutations in *MTOR* are most commonly associated with FCD type II.^5^, ^10^ Considering the pharmacological effect of sirolimus and elevated mTOR function, patients with FCD caused by a pathogenic variant of *MTOR* or other *MTOR*‐related genes respond more efficiently to sirolimus than those with condition not related to elevated mTOR function. Mutation analysis of brain tissue, which should ideally be performed prior to sirolimus treatment, is not always possible. FCD is mainly caused by a somatic mosaic mutation with low‐frequency mutant allele, which requires deep sequencing for detection, and samples of pathological brain tissues are needed for examination. In terms of practical use, the diagnosis of FCD type II would be a minimum requirement for selecting patients eligible for sirolimus.

Most patients with FCD experience their first seizure within the first 5 years.^1^ However, the age limit was decreased to accommodate children aged 6 years in the trial, with consideration of their average weight, which is about 20 kg. This can ensure that a starting dose of 1 mg/day can be administered safely, and children aged about 6 years can take tablets. Sirolimus is used for younger patients to prevent allograft rejection after kidney transplant^30^ or to treat complicated vascular anomalies.^31^ As indicated by the association between early‐onset or intractable frequent seizures and cognitive dysfunction in patients with FCD,^24^ additional studies about treatment for seizures in FCD patients aged younger than 6 years must be performed.

The current study has several limitations. That is, a placebo group was not included. The background characteristics of external control and sirolimus‐treated groups, including the proportion of patients with pathology‐proven FCD type II, differed. Moreover, the number of participants were limited, and the trial duration was relatively short. In total, 12 patients, including two from the preceding proof‐of‐concept study (manuscript submitted), received continuous sirolimus treatment in the extension trial. However, further investigations must be performed to confirm the efficacy and safety of short‐ and long‐term treatment with sirolimus.

In conclusion, the reduction of focal seizures did not meet the predetermined level of statistical significance. The safety profile of the drug was tolerable. The potential for a reduction of focal seizures over time merit further investigations in large and long‐term trials.

## Author Contributions

M. K. contributed to the conception of the study; M. K., A. Kada, J. T., and A. M. S. contributed to the design of the study; M. K. and A. Kada contributed to the analysis of data and drafting the text or preparing figures; A. Kada contributed to statistical analysis; H. S., J. T., E. N., Y. T., and T. A. contributed to enrollment of the patient and collection and analysis of clinical data; A. Kakita contributed to neuropathological diagnosis; N. M. and A. F. contributed to molecular analysis; A. M. S. contributed to the management of data; Y. I. contributed to the design of the external cohort study.

## Conflict of Interests

Y. T. received academic donation from Eisai. A. Kada, who is a member of the independent data monitoring committee of clinical trials, received personal fees from Bayer Yakuhin, Ltd. outside the submitted work. The other authors have no conflict of interest to disclose.

## Data Availability

De‐identified individual participant data (including data dictionaries) in addition to study protocols, statistical analyses, and informed consent forms will be made available upon publication to researchers who provide a methodologically sound proposal for the use of data. Proposals should be submitted to ktmthr@gmail.com.

## Supporting information


**Table S1.** Distribution of the causative genes in patients with each type of focal cortical dysplasia or hemimegalencephaly.

**Table S2.** Previous trials for epileptic seizures treated with mTOR inhibitors.

**Table S3.** All adverse events reported in patients receiving sirolimus.
