# Supplementary material for: Sirolimus for epileptic seizures associated with focal cortical dysplasia type II
Source: Ann Clin Transl Neurol. 2022 Jan 18;9(2):181–92. doi: 10.1002/acn3.51505 (PMC8862414; doi:10.1002/acn3.51505)
Supplement: Supplementary file 1 — Table S1. Distribution of the causative genes in patients with each type of focal cortical dysplasia or hemimegalencephaly. [file ACN3-9-181-s001.docx]

**Supplementary Table 1.** Distribution of the causative genes in patients with each type of focal cortical dysplasia or hemimegalencephaly.

| Gene | FCD | | | | HME | Reference |
| --- | --- | --- | --- | --- | --- | --- |
|  | I | IIa | IIb | unknown |  |  |
| *PIK3CA* |  | *1* |  |  | *17* | 1-6 |
| *PTEN* |  |  | 1 |  | 1 | 2, 7 |
| *AKT1* |  |  |  |  | 1 | 5 |
| *AKT3* | 1 | 2 |  |  | 8 | 1, 2, 4-6, 8, 9 |
| *TSC1* |  | 3 | 5 |  |  | 5, 6, 10, 11 |
| *TSC2* |  | 2 | 2 | 1 | 3 | 3, 5, 6, 10 |
| *RHEB* |  |  | 1 |  | 1 | 6 |
| *MTOR* |  | *26* | *28* | *2* | *10* | 1, 3, 5, 6, 12-19, our case |
| *DEPDC5* | *7* | *8* | *2* | *7* | *1* | 3, 5, 6, 16, 20-25 |
| *NPRL2* | 2 |  |  |  |  | 5, 24 |
| *NPRL3* |  | 5 |  | 1 |  | 24, 26 |
| *CNTNAP2* |  | *3* |  |  |  | 27 |
| *COL4A1* | *1* |  |  |  |  | 28 |

References

1. Lee JH, Huynh M, Silhavy JL, et al. De novo somatic mutations in components of the PI3K-AKT3-mTOR pathway cause hemimegalencephaly. Nat Genet. 2012;44(8):941-5.

2. Jansen LA, Mirzaa GM, Ishak GE, et al. PI3K/AKT pathway mutations cause a spectrum of brain malformations from megalencephaly to focal cortical dysplasia. Brain. 2015;138(Pt 6):1613-28.

3. D'Gama AM, Geng Y, Couto JA, et al. Mammalian target of rapamycin pathway mutations cause hemimegalencephaly and focal cortical dysplasia. Ann Neurol. 2015;77(4):720-5.

4. Alcantara D, Timms AE, Gripp K, et al. Mutations of *AKT3* are associated with a wide spectrum of developmental disorders including extreme megalencephaly. Brain. 2017;140(10):2610-22.

5. D'Gama AM, Woodworth MB, Hossain AA, et al. Somatic Mutations Activating the mTOR Pathway in Dorsal Telencephalic Progenitors Cause a Continuum of Cortical Dysplasias. Cell reports. 2017;21(13):3754-66.

6. Baldassari S, Ribierre T, Marsan E, et al. Dissecting the genetic basis of focal cortical dysplasia: a large cohort study. Acta Neuropathol. 2019;138(6):885-900.

7. Schick V, Majores M, Engels G, et al. Activation of Akt independent of PTEN and CTMP tumor-suppressor gene mutations in epilepsy-associated Taylor-type focal cortical dysplasias. Acta Neuropathol. 2006;112(6):715-25.

8. Poduri A, Evrony GD, Cai X, et al. Somatic Activation of AKT3 Causes Hemispheric Developmental Brain Malformations. Neuron. 2012;74(1):41-8.

9. Conti V, Pantaleo M, Barba C, et al. Focal dysplasia of the cerebral cortex and infantile spasms associated with somatic 1q21.1-q44 duplication including the AKT3 gene. Clin Genet. 2015;88(3):241-7.

10. Lim JS, Gopalappa R, Kim SH, et al. Somatic Mutations in *TSC1* and *TSC2* Cause Focal Cortical Dysplasia. Am J Hum Genet. 2017;100(3):454-72.

11. Hoelz H, Coppenrath E, Hoertnagel K, et al. Childhood-Onset Epileptic Encephalopathy Associated With Isolated Focal Cortical Dysplasia and a Novel TSC1 Germline Mutation. Clin EEG Neurosci. 2018;49(3):187-91.

12. Lim JS, Kim WI, Kang HC, et al. Brain somatic mutations in *MTOR* cause focal cortical dysplasia type II leading to intractable epilepsy. Nat Med. 2015;21(4):395-400.

13. Nakashima M, Saitsu H, Takei N, et al. Somatic Mutations in the *MTOR* gene cause focal cortical dysplasia type IIb. Ann Neurol. 2015;78(3):375-86.

14. Leventer RJ, Scerri T, Marsh AP, et al. Hemispheric cortical dysplasia secondary to a mosaic somatic mutation in MTOR. Neurology. 2015;84(20):2029-32.

15. Moller RS, Weckhuysen S, Chipaux M, et al. Germline and somatic mutations in the MTOR gene in focal cortical dysplasia and epilepsy. Neurol Genet. 2016;2(6):e118.

16. Mirzaa GM, Campbell CD, Solovieff N, et al. Association of *MTOR* Mutations With Developmental Brain Disorders, Including Megalencephaly, Focal Cortical Dysplasia, and Pigmentary Mosaicism. JAMA Neurol. 2016;73(7):836-45.

17. Hanai S, Sukigara S, Dai H, et al. Pathologic Active mTOR Mutation in Brain Malformation with Intractable Epilepsy Leads to Cell-Autonomous Migration Delay. Am J Pathol. 2017;187(5):1177-85.

18. Griffin NG, Cronin KD, Walley NM, et al. Somatic uniparental disomy of Chromosome 16p in hemimegalencephaly. Cold Spring Harb Mol Case Stud. 2017;3(5).

19. Xu Q, Uliel-Sibony S, Dunham C, et al. mTOR inhibitors as a new therapeutic strategy in treatment resistant epilepsy in hemimegalencephaly: a case report. J Child Neurol. 2019;34(3):132-8.

20. Scheffer IE, Heron SE, Regan BM, et al. Mutations in mammalian target of rapamycin regulator *DEPDC5* cause focal epilepsy with brain malformations. Ann Neurol. 2014;75(5):782-7.

21. Baulac S, Ishida S, Marsan E, et al. Familial focal epilepsy with focal cortical dysplasia due to DEPDC5 mutations. Ann Neurol. 2015;77(4):675-83.

22. Carvill GL, Crompton DE, Regan BM, et al. Epileptic spasms are a feature of DEPDC5 mTORopathy. Neurol Genet. 2015;1(2):e17.

23. Ricos MG, Hodgson BL, Pippucci T, et al. Mutations in the mammalian target of rapamycin pathway regulators NPRL2 and NPRL3 cause focal epilepsy. Ann Neurol. 2016;79(1):120-31.

24. Weckhuysen S, Marsan E, Lambrecq V, et al. Involvement of GATOR complex genes in familial focal epilepsies and focal cortical dysplasia. Epilepsia. 2016;57(6):994-1003.

25. Ribierre T, Deleuze C, Bacq A, et al. Second-hit mosaic mutation in mTORC1 repressor DEPDC5 causes focal cortical dysplasia-associated epilepsy. J Clin Invest. 2018;128(6):2452-8.

26. Sim JC, Scerri T, Fanjul-Fernandez M, et al. Familial cortical dysplasia caused by mutation in the mammalian target of rapamycin regulator NPRL3. Ann Neurol. 2016;79(1):132-7.

27. Strauss KA, Puffenberger EG, Huentelman MJ, et al. Recessive symptomatic focal epilepsy and mutant contactin-associated protein-like 2. N Engl J Med. 2006;354(13):1370-7.

28. Yoneda Y, Haginoya K, Kato M, et al. Phenotypic Spectrum of *COL4A1* Mutations: Porencephaly to Schizencephaly. Ann Neurol. 2013;73(1):48-57.
