# Supplementary material for: Sirolimus for epileptic seizures associated with focal cortical dysplasia type II
Source: Ann Clin Transl Neurol. 2022 Jan 18;9(2):181–92. doi: 10.1002/acn3.51505 (PMC8862414; doi:10.1002/acn3.51505)
Supplement: Supplementary file 2 — Table S2. Previous trials for epileptic seizures treated with mTOR inhibitors. [file ACN3-9-181-s003.docx]

**Supplementary Table 2.** Previous trials for epileptic seizures treated with mTOR inhibitors.

| Disease | Medicine | Study design | No. of patients with seizures (range of age in the study) | Dose or target trough levels of the drug | Duration of treatment | Efficacy for seizure (decreased/not changed/worsened) | Ref |
| --- | --- | --- | --- | --- | --- | --- | --- |
| TSC | Siro | Case report | 1 (10 years) | 0.15mg/kg/day | 10 months | A dramatic reduction in seizure frequency (seizure clusters stopped; however, 1 to 5 brief seizures (< 2 minutes) continued daily) | 1 |
| TSC | Ever | Prospective, open-label, phase 1–2 study | 16 (3–34 years) | 3.0 mg/m^2^ | 6 months | (9/6/1) | 2 |
| TSC | Ever | Case report | 1 (10 years) | 4.5mg/m^2^/day | 12 months | Cessation of seizures | 3 |
| TSC | Ever | Prospective, multicenter, open-label, phase I/II clinical trial. | 20 (2–21 years) | Initial dose: 2.5 or 5 mg (5mg/m^2^/day), then adjusted for a target range between 5 and 15ng/ml | 4-week titration and an 8-week maintenance | Seizure frequency was reduced by more than 50% in 12 of 20 subjects. Overall, seizures were reduced in 17 of the 20 by a median reduction of 73%. (15/5/0) | 4 |
| TSC | Ever | Compassionate use trial | 7 (2–12 years) | 3 mg/m^2^ /day (titrated to target blood trough levels of 5–15 ng/mL) | 36 weeks | (4/2/0) | 5 |
| TSC | Siro 6, ever 1 | Open-label single-center series | 7 (3–14 years) | Sirolimus 1–5mg, everolimus 5mg at 14 years of age | 6 - 38 months | One patient had more than 90% reduction, 4 had 50%-90% reduction, and 2 had less than 50% reduction. | 6 |
| TSC | Ever | Open-label phase II study | 26 (3–34 years) | 3 mg/m^2^ /day (titrated to target blood trough levels of 5–15 ng/mL) | more than 5 years | Daily seizures was reduced from 7 out of 26 (26.9%) patients at baseline to 2 out of 18 (11.1%) patients at month 60. | 7 |
| TSC | Ever | Phase 3, randomised, double-blind, placebo-controlled study | 117 for low- and 130 for high-exposure (2–65 years) | 3–7 ng/mL for low exposure and 9–15 ng/mL for high exposure | 6-week titration period, and 12-week maintenance period | The response rate was 15·1% with placebo compared with 28·2% for low-exposure and 40·0% for high-exposure. | 8 |
| TSC | Ever | Post-hoc analysis of the phase 3 EXIST-3 trial | 96 for low-exposure and 107 for high-exposure (2–5 years or 6–17 years) | 3–7 ng/mL for low exposure and 9–15 ng/mL for high exposure | More than 48 weeks after EXIST-3 trial | The response rate was 17.6% for placebo vs 30.3% for low-exposure vs 59.5% for high-exposure in the younger subgroup and 12.9% vs 27.0% vs 30.0% in the older subgroup. | 9 |
| HME | Siro | Case report | 1 (3 months) | 0.3 mg (1 mg/m^2^) and increased to 0.35 mg (4.4 ng/mL of the highest serum level) | 2.5 months before the hemispherectomy | After 1 week of treatment, there was >50% reduction in seizures and total seizure burden, and after 2 weeks, development improved, resulting in deferral of surgery by 2.5 months with an increased body weight. | 10 |

Eve, everolimus; HME, hemimegalencephaly; Siro, sirolimus, TSC, tuberous sclerosis complex

References

1. Muncy J, Butler IJ, Koenig MK. Rapamycin reduces seizure frequency in tuberous sclerosis complex. J Child Neurol. 2009;24(4):477.

2. Krueger DA, Care MM, Holland K, et al. Everolimus for subependymal giant-cell astrocytomas in tuberous sclerosis. N Engl J Med. 2010;363(19):1801-11.

3. Perek-Polnik M, Jozwiak S, Jurkiewicz E, Perek D, Kotulska K. Effective everolimus treatment of inoperable, life-threatening subependymal giant cell astrocytoma and intractable epilepsy in a patient with tuberous sclerosis complex. Eur J Paediatr Neurol. 2012;16(1):83-5.

4. Krueger DA, Wilfong AA, Holland-Bouley K, et al. Everolimus treatment of refractory epilepsy in tuberous sclerosis complex. Ann Neurol. 2013;74(5):679-87.

5. Wiegand G, May TW, Ostertag P, Boor R, Stephani U, Franz DN. Everolimus in tuberous sclerosis patients with intractable epilepsy: a treatment option? Eur J Paediatr Neurol. 2013;17(6):631-8.

6. Cardamone M, Flanagan D, Mowat D, Kennedy SE, Chopra M, Lawson JA. Mammalian target of rapamycin inhibitors for intractable epilepsy and subependymal giant cell astrocytomas in tuberous sclerosis complex. J Pediatr. 2014;164(5):1195-200.

7. Franz DN, Agricola K, Mays M, et al. Everolimus for subependymal giant cell astrocytoma: 5-year final analysis. Ann Neurol. 2015;78(6):929-38.

8. French JA, Lawson JA, Yapici Z, et al. Adjunctive everolimus therapy for treatment-resistant focal-onset seizures associated with tuberous sclerosis (EXIST-3): a phase 3, randomised, double-blind, placebo-controlled study. Lancet. 2016;388(10056):2153-63.

9. Curatolo P, Franz DN, Lawson JA, et al. Adjunctive everolimus for children and adolescents with treatment-refractory seizures associated with tuberous sclerosis complex: post-hoc analysis of the phase 3 EXIST-3 trial. Lancet Child Adolesc Health. 2018;2(7):495-504.

10. Xu Q, Uliel-Sibony S, Dunham C, et al. mTOR inhibitors as a new therapeutic strategy in treatment resistant epilepsy in hemimegalencephaly: a case report. J Child Neurol. 2019;34(3):132-8.
