# Supplementary material for: Sirolimus for epileptic seizures associated with focal cortical dysplasia type II
Source: Ann Clin Transl Neurol. 2022 Jan 18;9(2):181–92. doi: 10.1002/acn3.51505 (PMC8862414; doi:10.1002/acn3.51505)
Supplement: Supplementary file 3 — Table S3. All adverse events reported in patients receiving sirolimus. [file ACN3-9-181-s002.docx]

**Supplementary Table 3**. All adverse events reported in patients receiving sirolimus

Category Total Grade 1 Grade 2 Grade 3

Total 16 14 9 3

Infections 9 7 4 1

Conjunctivitis 1 0 1 0

Gastroenteritis 2 1 0 1

Herpes zoster 1 0 1 0

Sty 1 0 1 0

Influenza 1 1 0 0

Pharyngitis 6 5 1 0

Upper respiratory infection 1 0 1 0

Viral upper respiratory infection 1 1 0 0

Mycoplasma bronchitis 1 0 1 0

Blood and lymphatic system disorders 1 1 0 0

Anemia 1 1 0 0

Psychiatric disorders 1 0 1 0

Insomnia 1 0 1 0

Nervous system disorders 6 3 2 2

Dizziness 1 1 0 0

Epileptic seizure 1 0 0 1

Headache 2 1 1 0

Somnolence 1 1 1 0

Status epilepticus 1 0 0 1

Balance disorder 1 1 0 0

Eye disorders 1 0 1 0

Blepharitis 1 0 1 0

Chalazion 1 0 1 0

Respiratory, thoracic, and mediastinal disorders 3 3 2 0

Cough 1 1 0 0

Epistaxis 1 1 0 0

Pneumonitis 1 0 1 0

Nasal drip 2 1 1 0

Gastrointestinal disorders 11 8 4 0

Constipation 1 0 1 0

Diarrhea 1 1 0 0

Nausea 1 1 0 0

Stomatitis 11 7 4 0

Vomiting 1 1 0 0

Skin and subcutaneous tissue disorders 7 6 2 0

Acne 1 1 0 0

Dermatitis 2 2 0 0

Allergic dermatitis 1 1 0 0

Dry skin 1 1 0 0

Eczema 1 1 0 0

Miliaria 1 0 1 0

Urticaria 1 0 1 0

Asteatosis 1 1 0 0

Renal and urinary disorders 1 1 0 0

Proteinuria 1 1 0 0

General disorders and administration site conditions 1 1 0 0

Fever 1 1 0 0

Investigations 1 1 0 0

Increased alanine aminotransferase levels 1 1 0 0

Increased aspartate aminotransferase levels 1 1 0 0

Injury, poisoning, and procedural complications 1 0 0 1

Skin tear 1 0 0 1
